# Supplementary material for: A near chromosome-level genome assembly of a ghost moth (Lepidoptera, Hepialidae)
Source: Sci Data. 2024 Oct 16;11:1139. doi: 10.1038/s41597-024-03783-2 (PMC11484951; doi:10.1038/s41597-024-03783-2)
Supplement: Supplementary file 2 — HiFi sequence report [file 41597_2024_3783_MOESM2_ESM.pdf]

Report for dataset Lep 89533-Cell2 (all samples)

Dataset 6b28562e-425d-4469-8bab-271d2e23fb19

Summary

**Name**	Lep 89533-Cell2 (all samples)
**Created At**	2023-07-16 14:20:43.187
**Number of Records**	4995450
**Total Length**	68367713586
**Movie ID**	m84100_230715_010611_s2
**ICS Version**	12.0.0.179648
**Well Sample**	Lep 89533
**Biological Sample**	Lep 89533
**Barcode Name**	default

CCS Processing

Summary

**ZMWs input**	20,097,756
**ZMWs pass filters**	5,672,860
**ZMWs fail filters**	14,424,896
**ZMWs shortcut filters**	0
**ZMWs with tandem repeats**	98,199
**Below SNR threshold**	202,210
**Median length filter**	0
**Lacking full passes**	13,593,403
**Heteroduplex insertions**	149,005
**Coverage drops**	5,483
**Insufficient draft cov**	100,535
**Draft too different**	1
**Draft generation error**	319,437
**Draft above --max-length**	0
**Draft below --min-length**	167
**Reads failed polishing**	18,816
**Empty coverage windows**	20,693
**CCS did not converge**	9,954
**CCS adapter concatenation**	1
**CCS adapter palindrome**	81
**CCS adapter residue**	141
**CCS below minimum RQ**	4,969
**Unknown error**	0
**ZMWs missing adapters**	387,377

Adapter Report

Summary

**Adapter Dimers (0-10bp) %**	0
**Short Inserts (11-100bp) %**	0
**Local Base Rate**	1.79

CCS Analysis Report

Summary

**HiFi Reads**	5,194,461
**HiFi Yield (bp)**	71,087,217,156
**HiFi Read Length (mean, bp)**	13,685
**HiFi Read Length (median, bp)**	13,037
**HiFi Read Length N50 (bp)**	13,619
**HiFi Read Quality (median)**	Q31
**HiFi Read Quality (median)**	31
**HiFi Number of Passes (mean)**	8

HiFi Read Length Summary

Read Length (bp)	Reads	Reads (%)	Yield (bp)	Yield (%)
0	5194461	100	71087217156	100
5,000	5190332	100	71076141522	100
10,000	5149082	99	70696834176	99
15,000	1392291	27	24350856353	34
20,000	183627	4	3948325916	6
25,000	2845	0	74239402	0
30,000	93	0	3100495	0
35,000	23	0	889742	0
40,000	5	0	221033	0

HiFi Read Quality Summary

Read Quality (Phred)	Reads	Reads (%)	Yield (bp)	Yield (%)
Q20	5194461	100	71087217156	100
Q30	2772323	53	36882379708	52
Q40	672353	13	8281887109	12
Q50	59480	1	666842046	1

HiFi Read Length Distribution

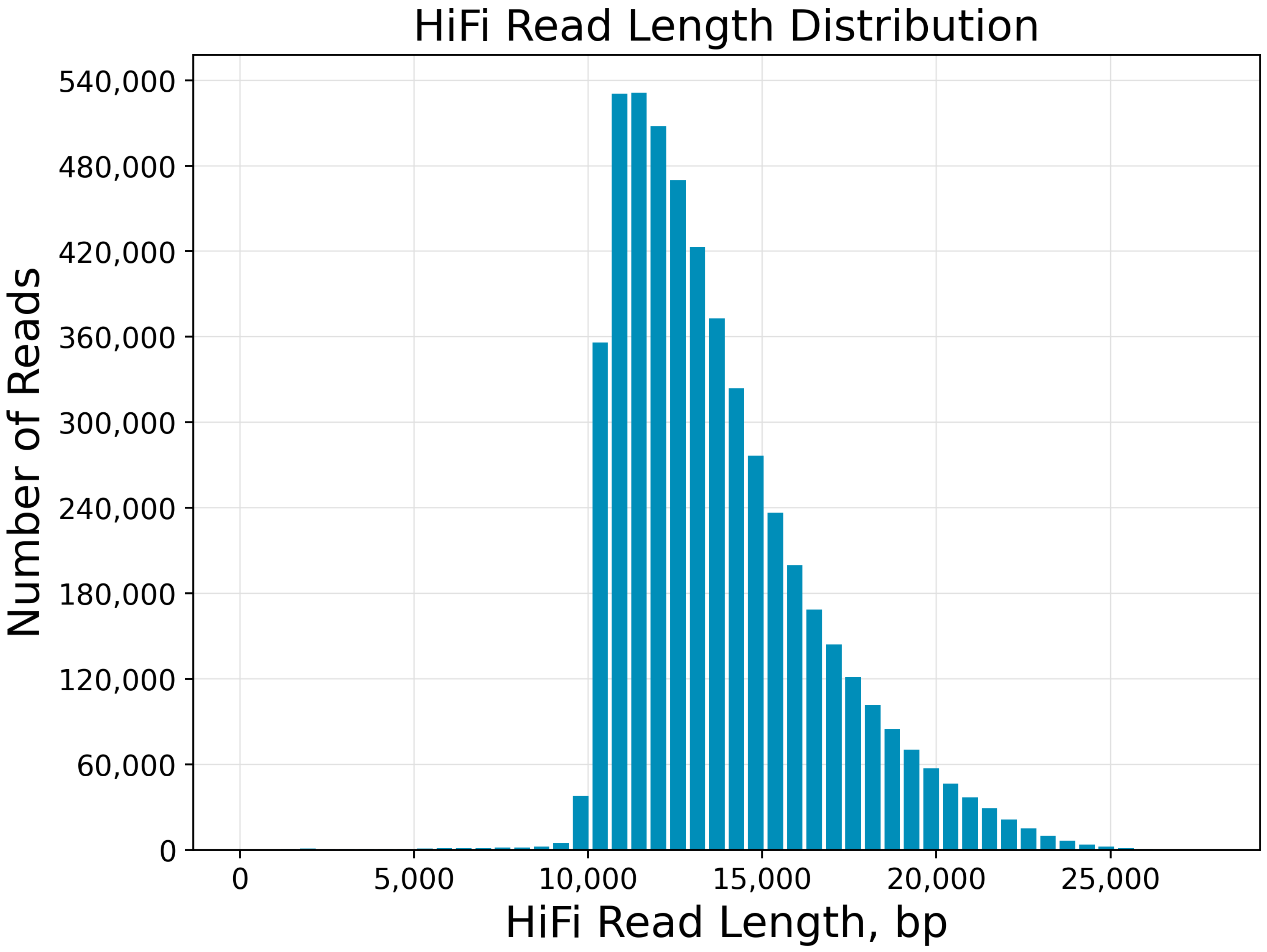

Yield by HiFi Read Length

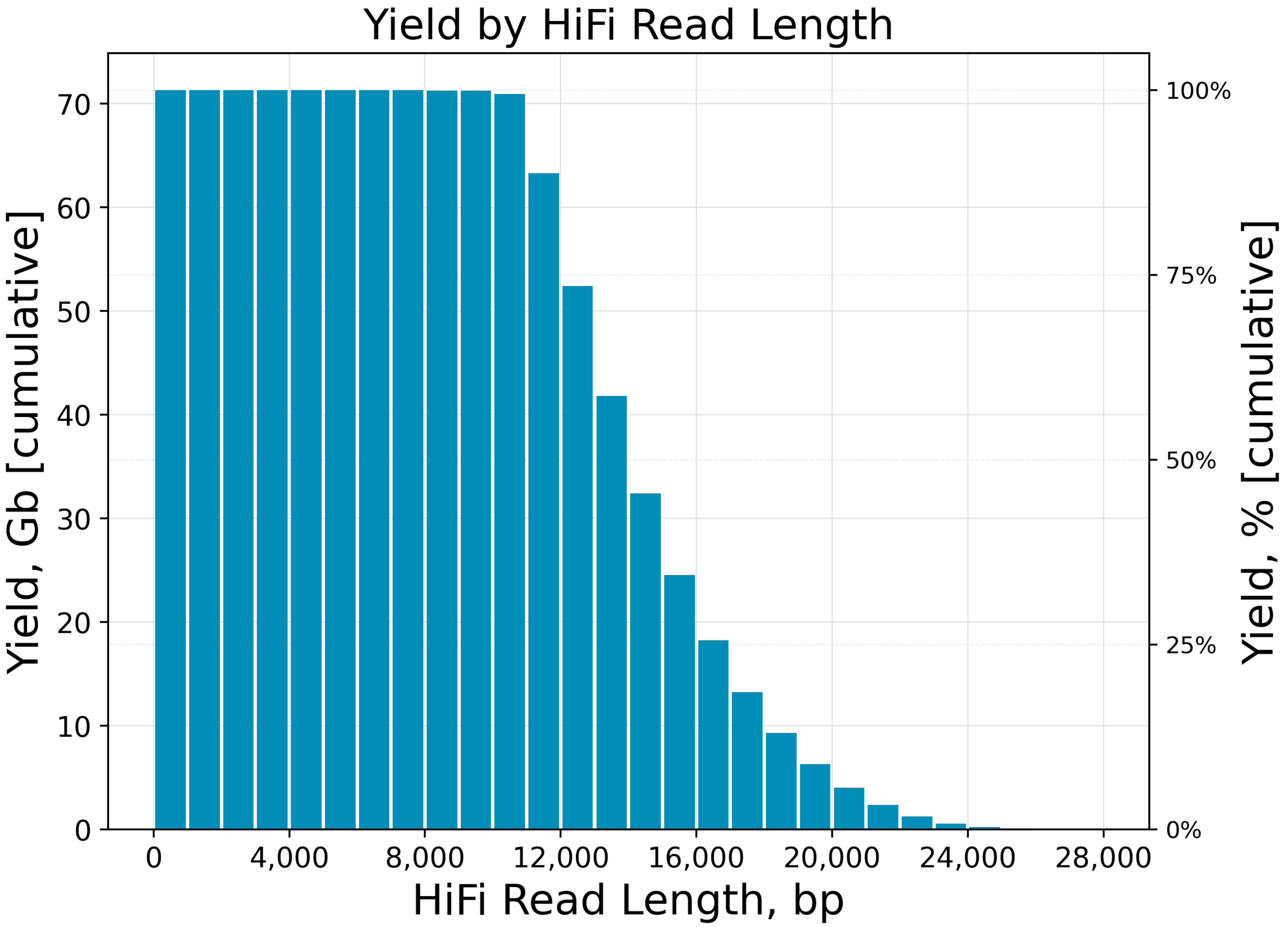

Read Length Distribution

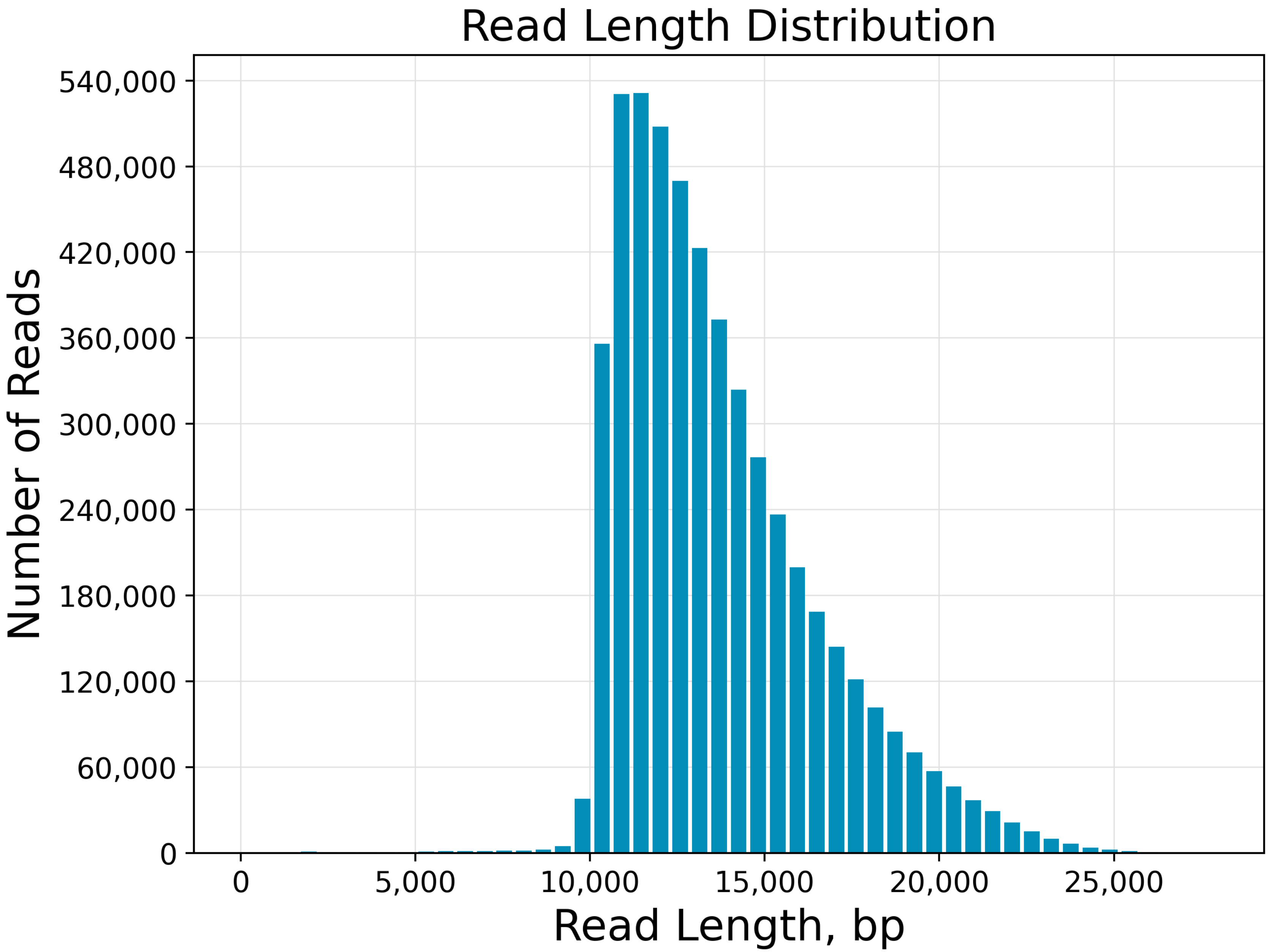

Number of Passes

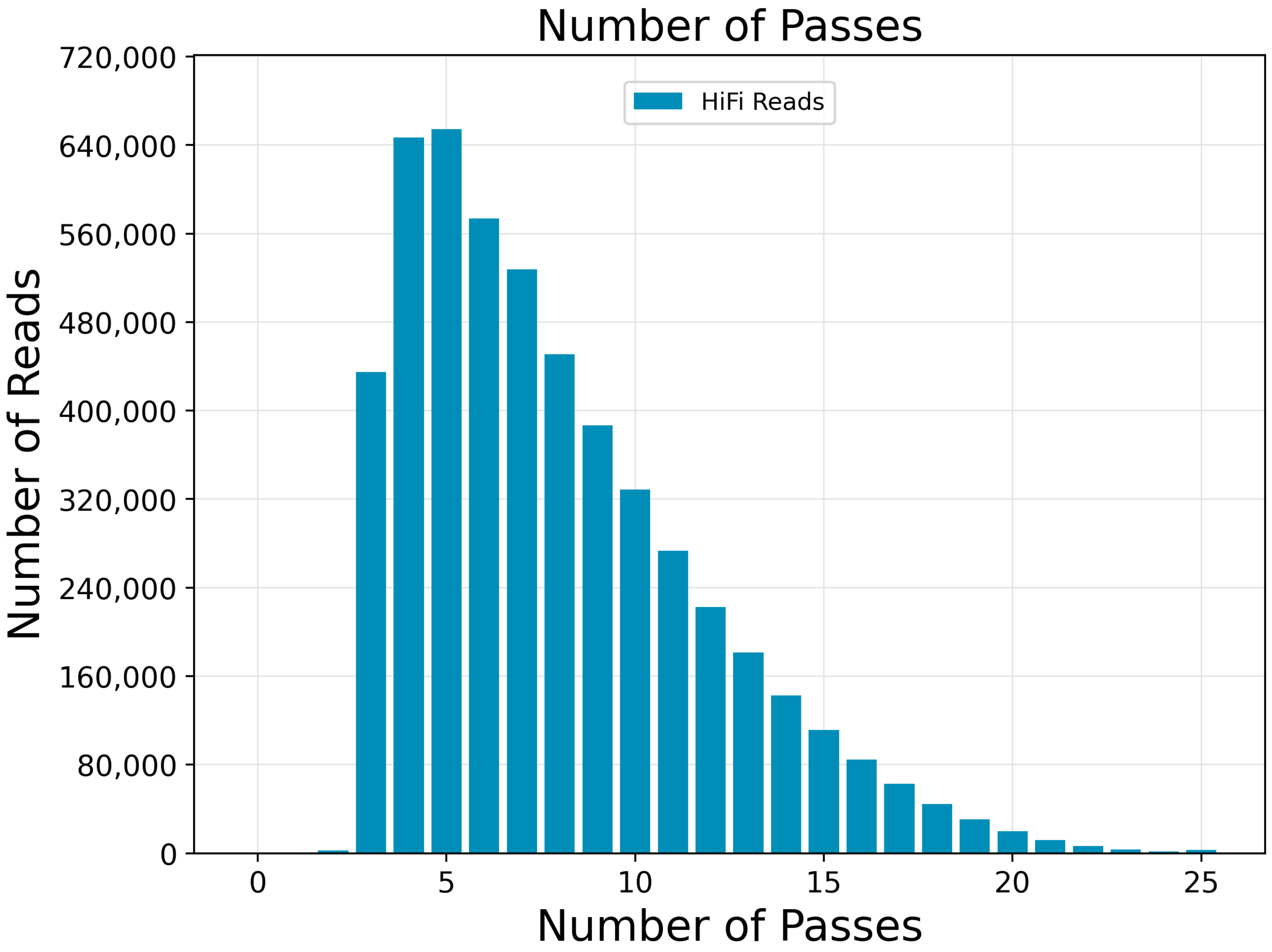

Read Quality Distribution

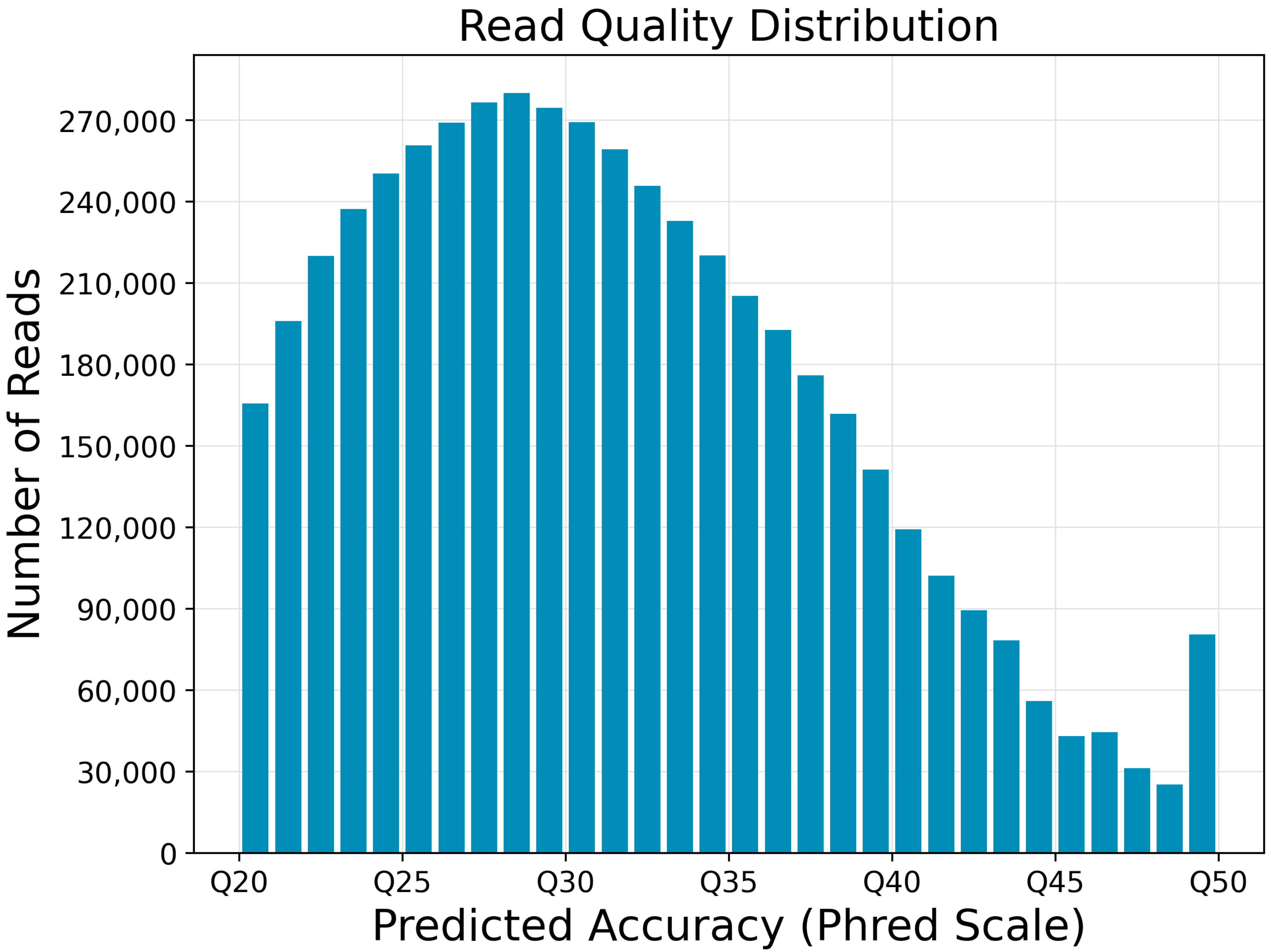

Predicted Accuracy vs. Read Length

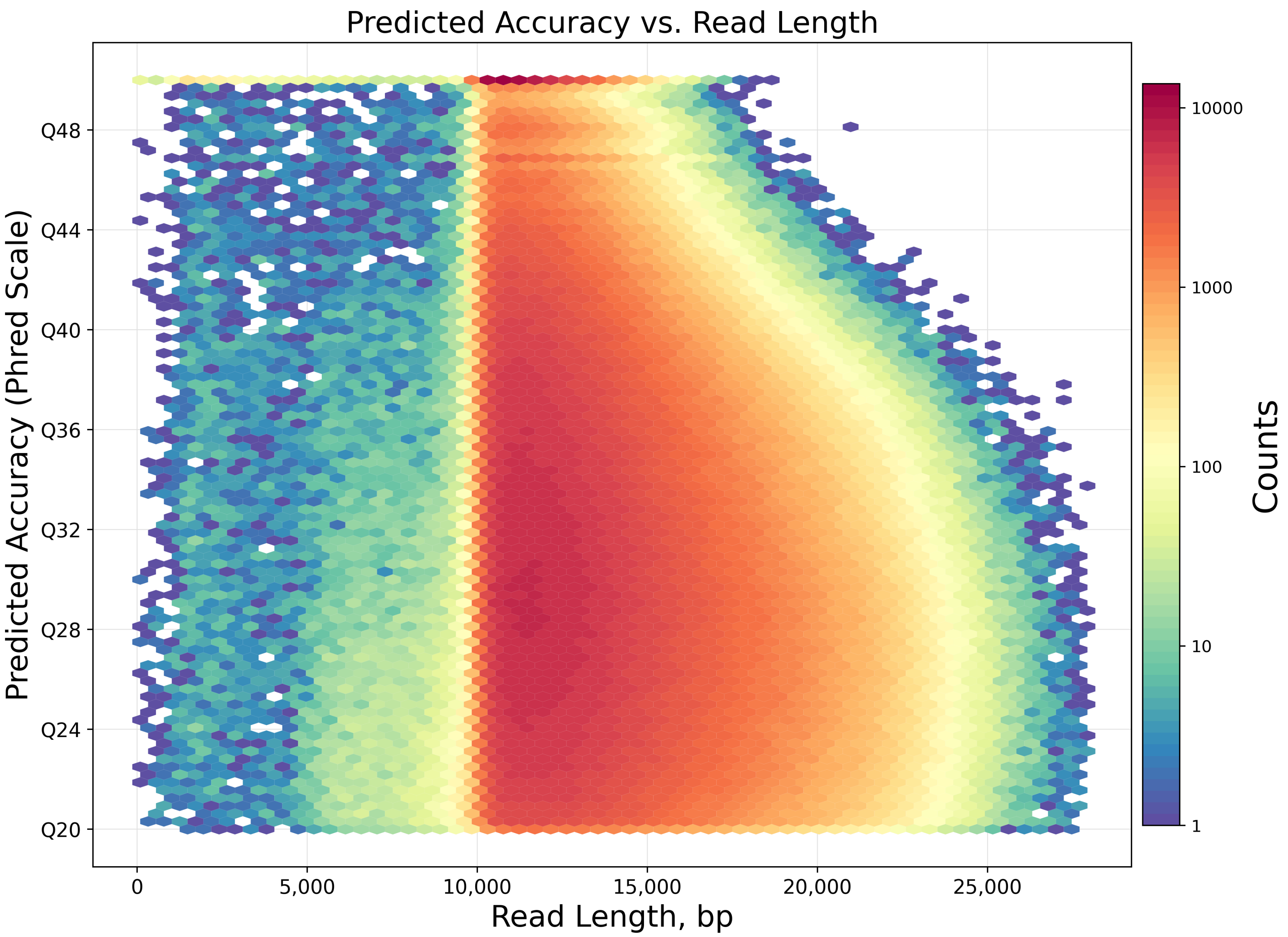

Control Report

Summary

**Number of Control Reads**	660
**Control Read Length Mean**	46,256
**Control Read Concordance Mean**	0.90
**Control Read Concordance Mode**	0.91

Control Polymerase RL

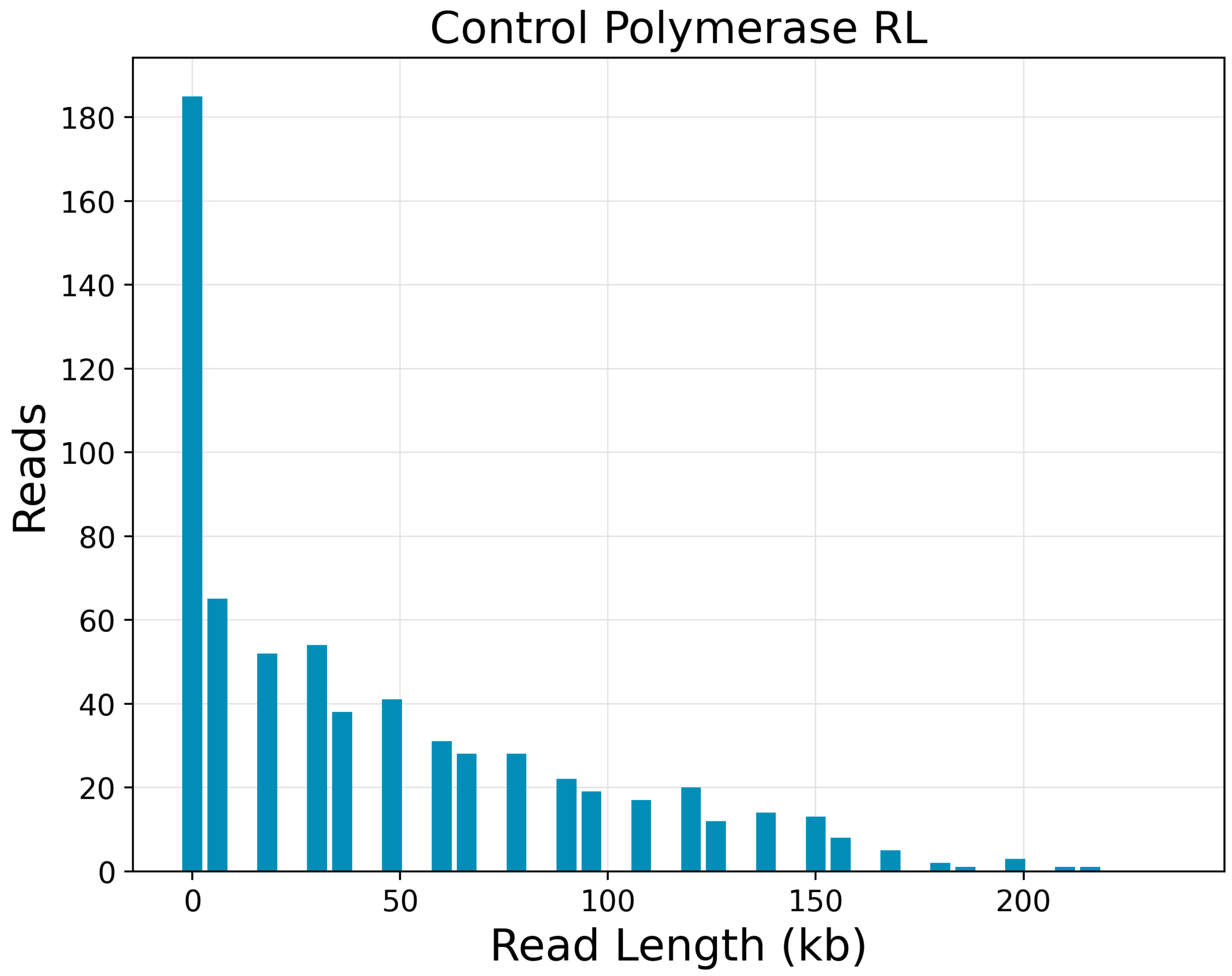
Control Polymerase RL
Reads
Read Length (kb)
0
20
40
60
80
100
120
140
160
180
0
50
100
150
200

Control Concordance

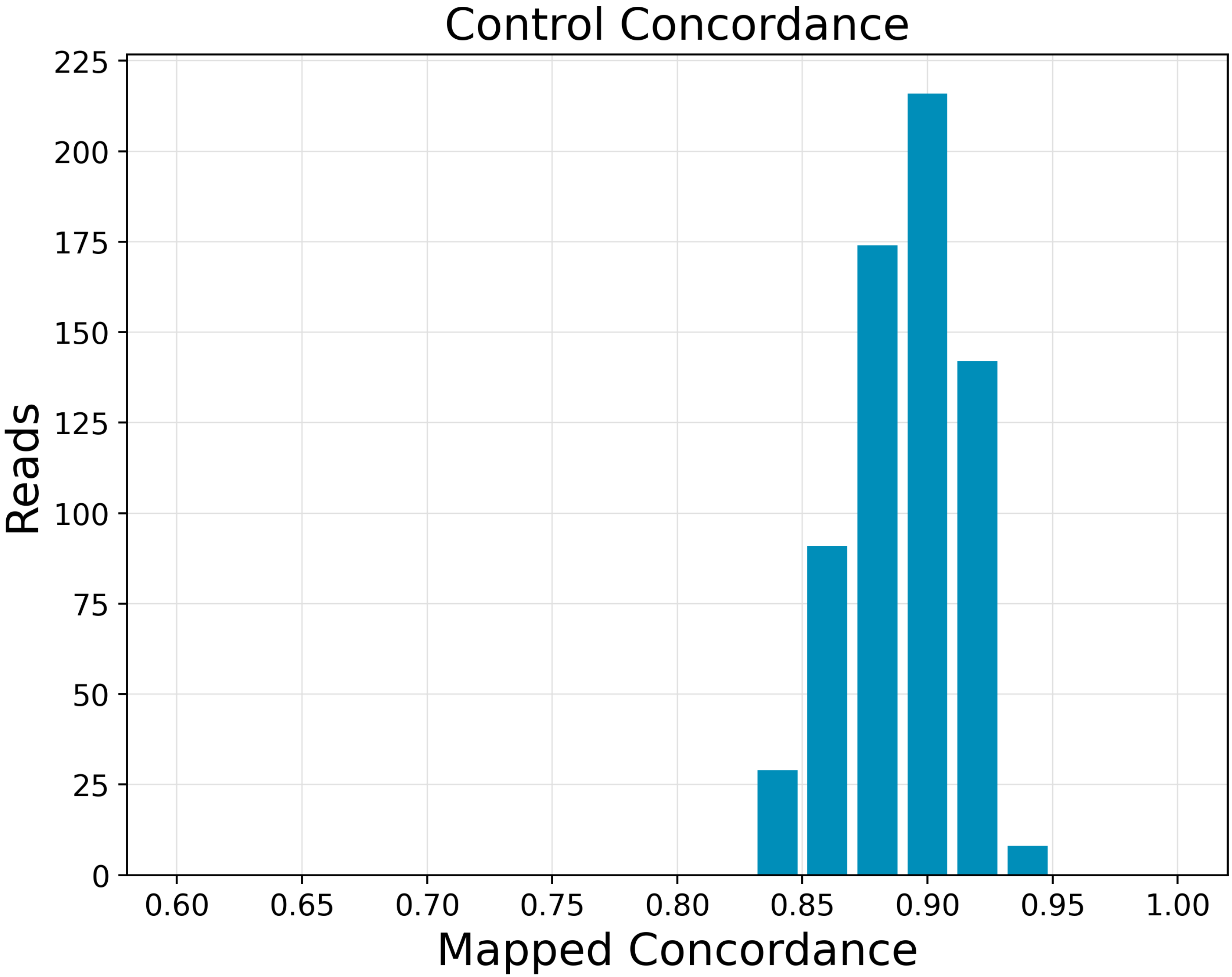
Control Concordance
Reads
Mapped Concordance
0
25
50
75
100
125
150
175
200
225
0.60
0.65
0.70
0.75
0.80
0.85
0.90
0.95
1.00

5mC CpG Report

CpG Methylation in Reads

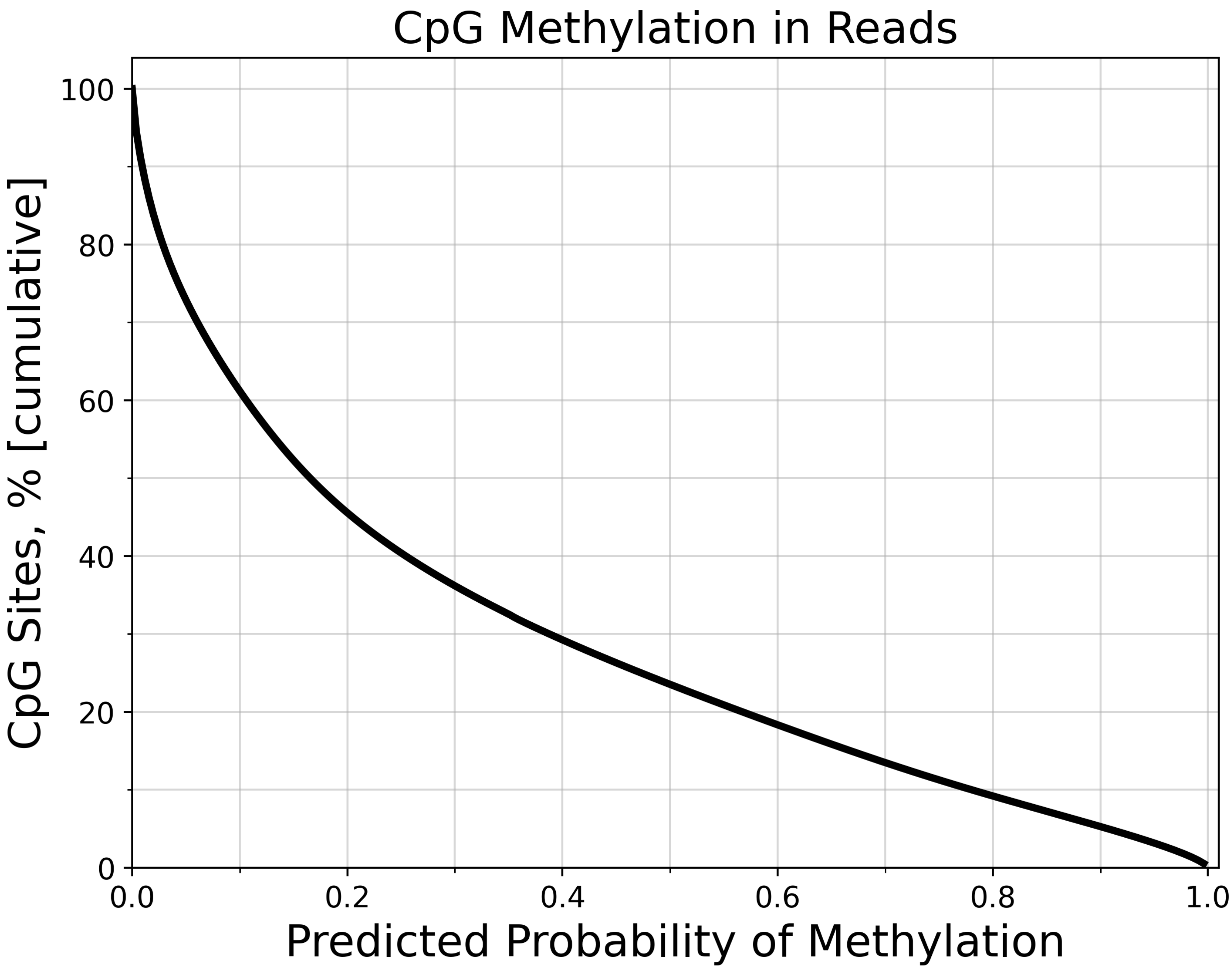
CpG Methylation in Reads
CpG Sites, % [cumulative]
Predicted Probability of Methylation
0
20
40
60
80
100
0.0
0.2
0.4
0.6
0.8
1.0

CpG Methylation in Reads (Histogram)

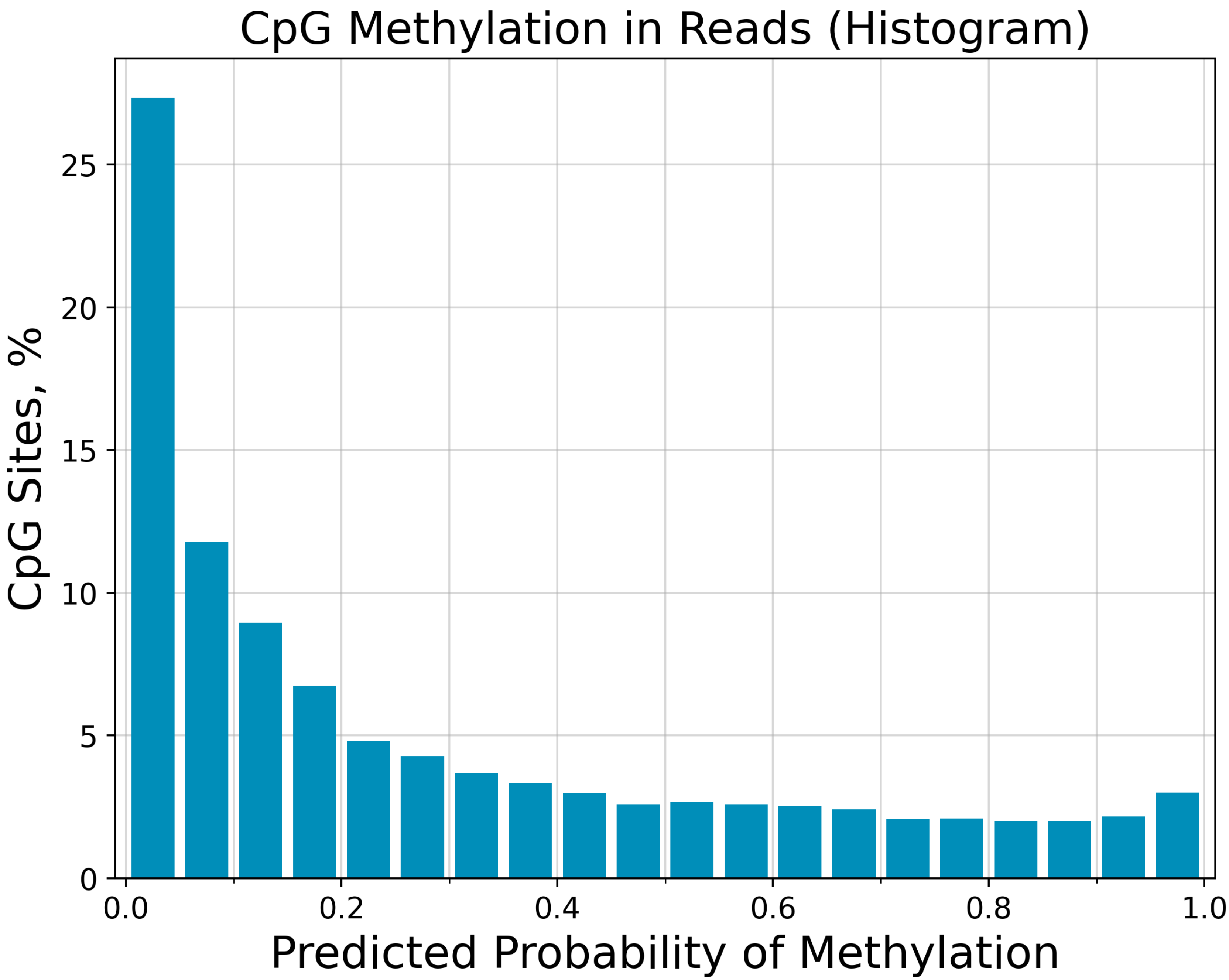
CpG Methylation in Reads (Histogram)
CpG Sites, %
Predicted Probability of Methylation
0
5
10
15
20
25
0.0
0.2
0.4
0.6
0.8
1.0

Loading Report

Summary

**Productive ZMWs**	16,777,216
**Productivity 0**	3,927,778
**Productivity 1**	20,205,425
**Productivity 2**	1,032,621

Loading Statistics

Collection Context	Productive ZMWs	Productivity 0	(%)	Productivity 1	(%)	Productivity 2	(%)	Loading type
m84100_230715_010611_s2	16777216	3927778	15.61	20205425	80.29	1032621	4.10	Workflow_Kestrel.py

HQ Region Filtering Evaluation

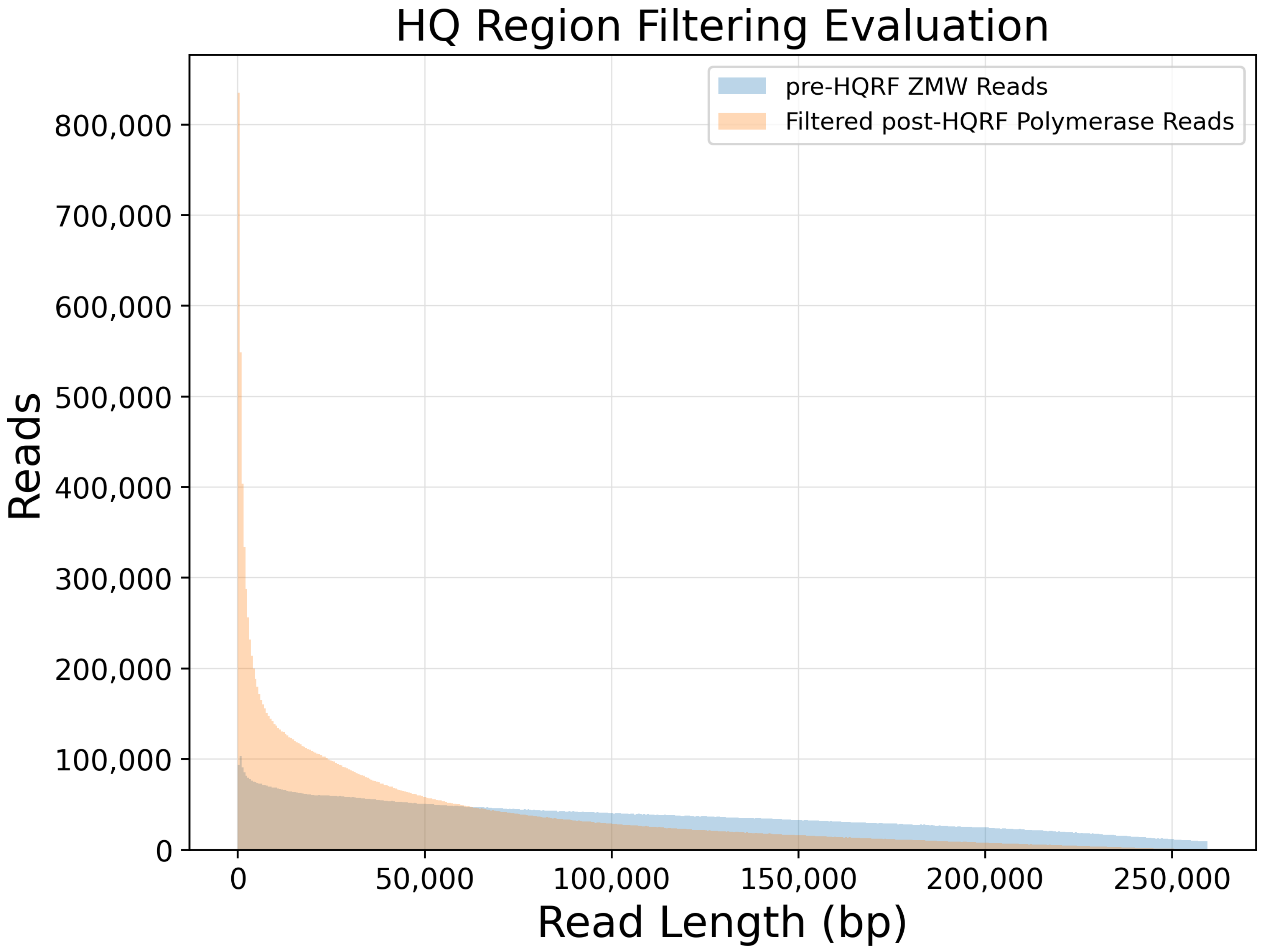

Raw Data Report

Summary

**Polymerase Read Bases**	1,042,893,191,193
**Polymerase Reads**	20,204,765
**Polymerase Read Length (mean)**	51,616
**Polymerase Read N50**	105,250
**Longest Subread Length (mean)**	17,059
**Longest Subread N50**	22,250
**Unique Molecular Yield**	320,789,413,888

Polymerase Read Length

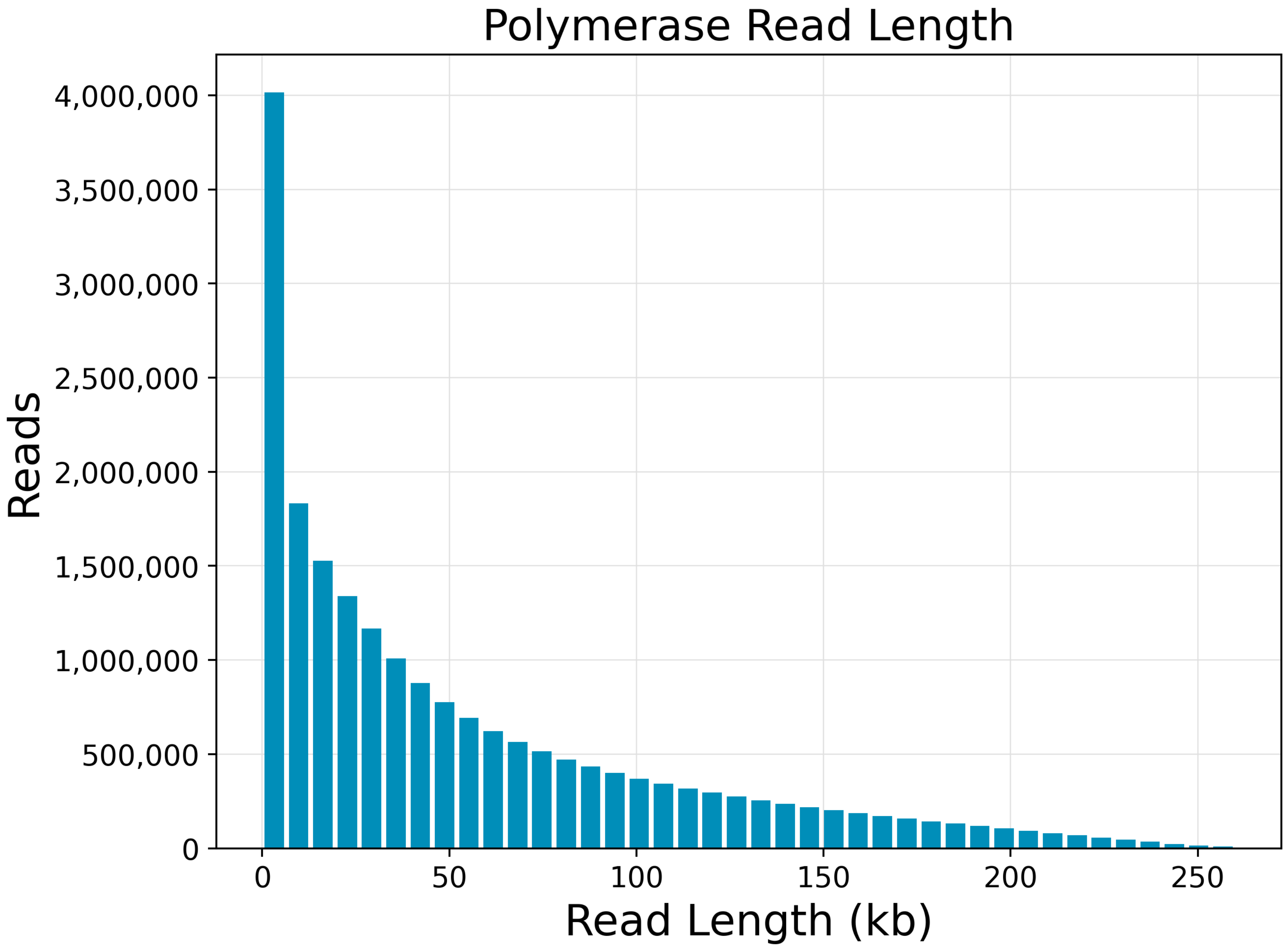

Longest Subread Length Versus Polymerase Read Length

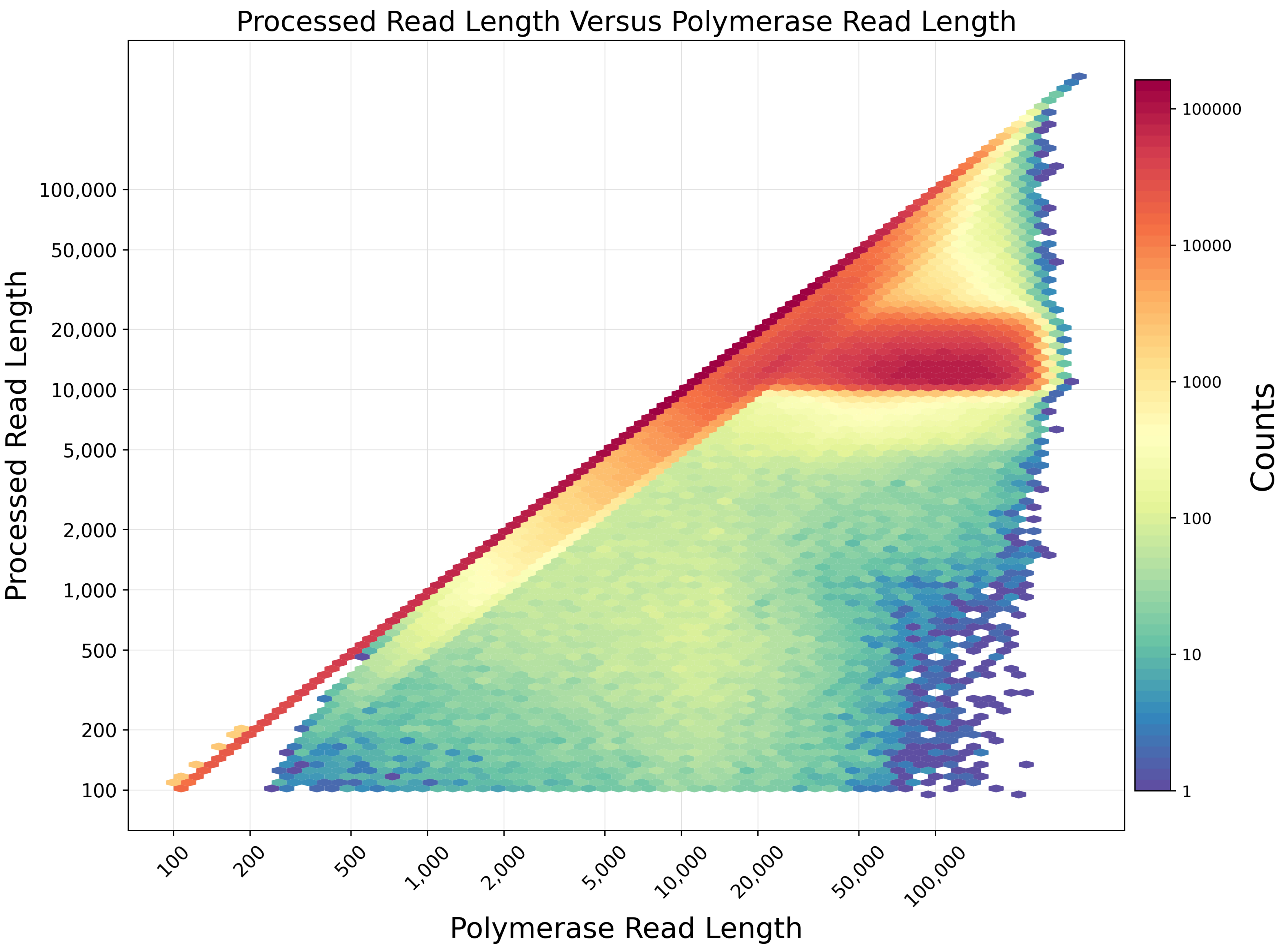

Base Yield Density

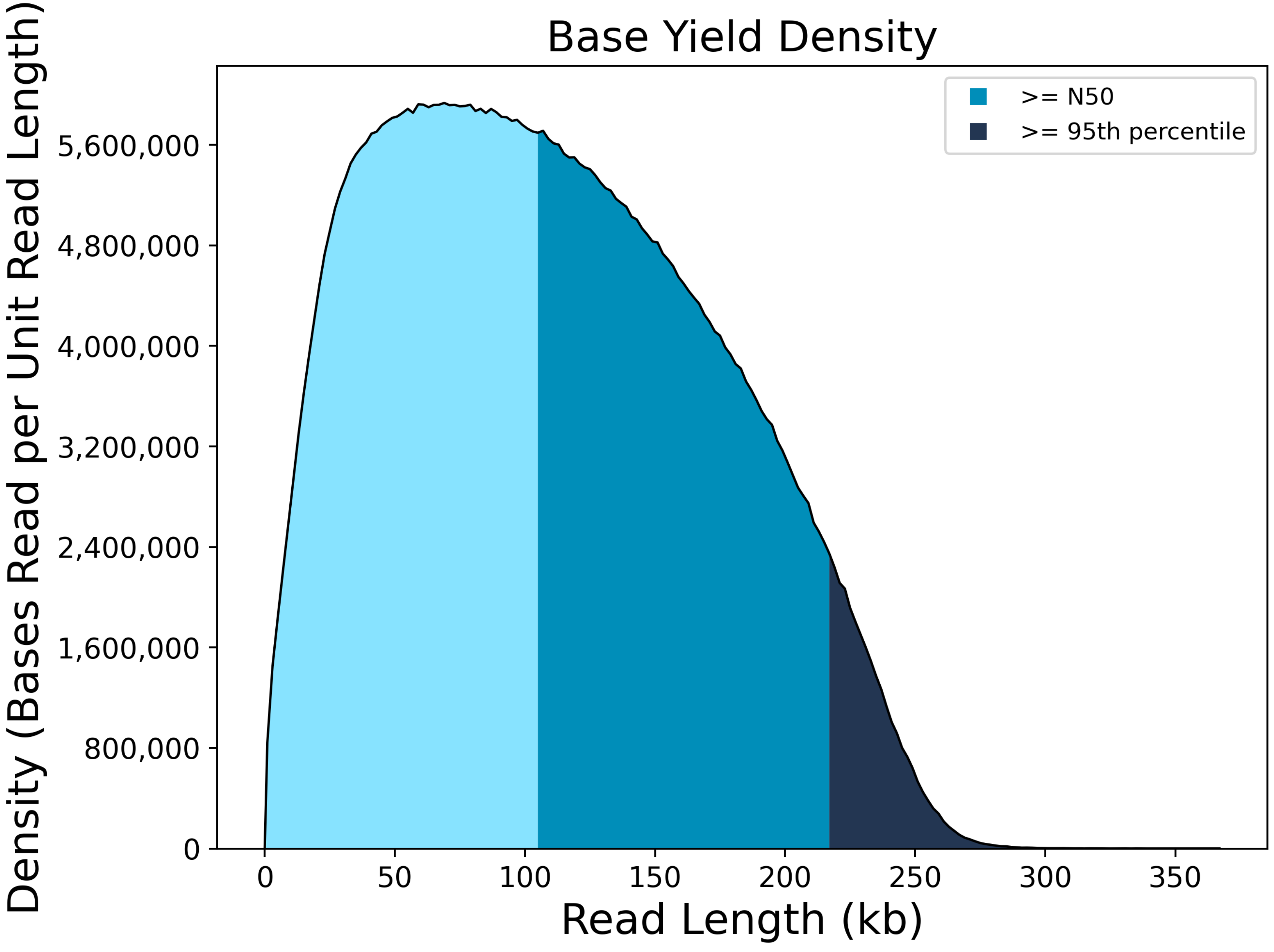

Barcodes

Summary

**Unique Barcodes**	1
**Barcoded HiFi Reads**	4,995,450
**Unbarcoded HiFi Reads**	199,011
**Barcoded HiFi Reads (%)**	0.961687844032326
**Barcoded HiFi Yield (bp)**	68,367,713,586
**Unbarcoded HiFi Yield (bp)**	2,719,503,570
**Barcoded HiFi Yield (%)**	0.9617441267389595
**Mean HiFi Reads per Barcode**	4,995,450
**Max. HiFi Reads per Barcode**	4,995,450
**Min. HiFi Reads per Barcode**	4,995,450
**Barcoded HiFi Read Length (mean, bp)**	13,685
**Unbarcoded HiFi Read Length (mean, bp)**	13,665

Barcode Data

Sample Name	Barcode	Barcode Quality	HiFi Reads	HiFi Read Length (mean, bp)	HiFi Read Quality (mean, QV)	HiFi Yield (bp)	Polymerase Read Length (mean, bp)	Polymerase Yield (bp)
Lep 89533	default--default	93.0	4995450	13685	Q28	68367713586	null	0
No Name	Not Barcoded	0.0	199011	13665	Q24	2719503570	null	0

Number Of Reads Per Barcode

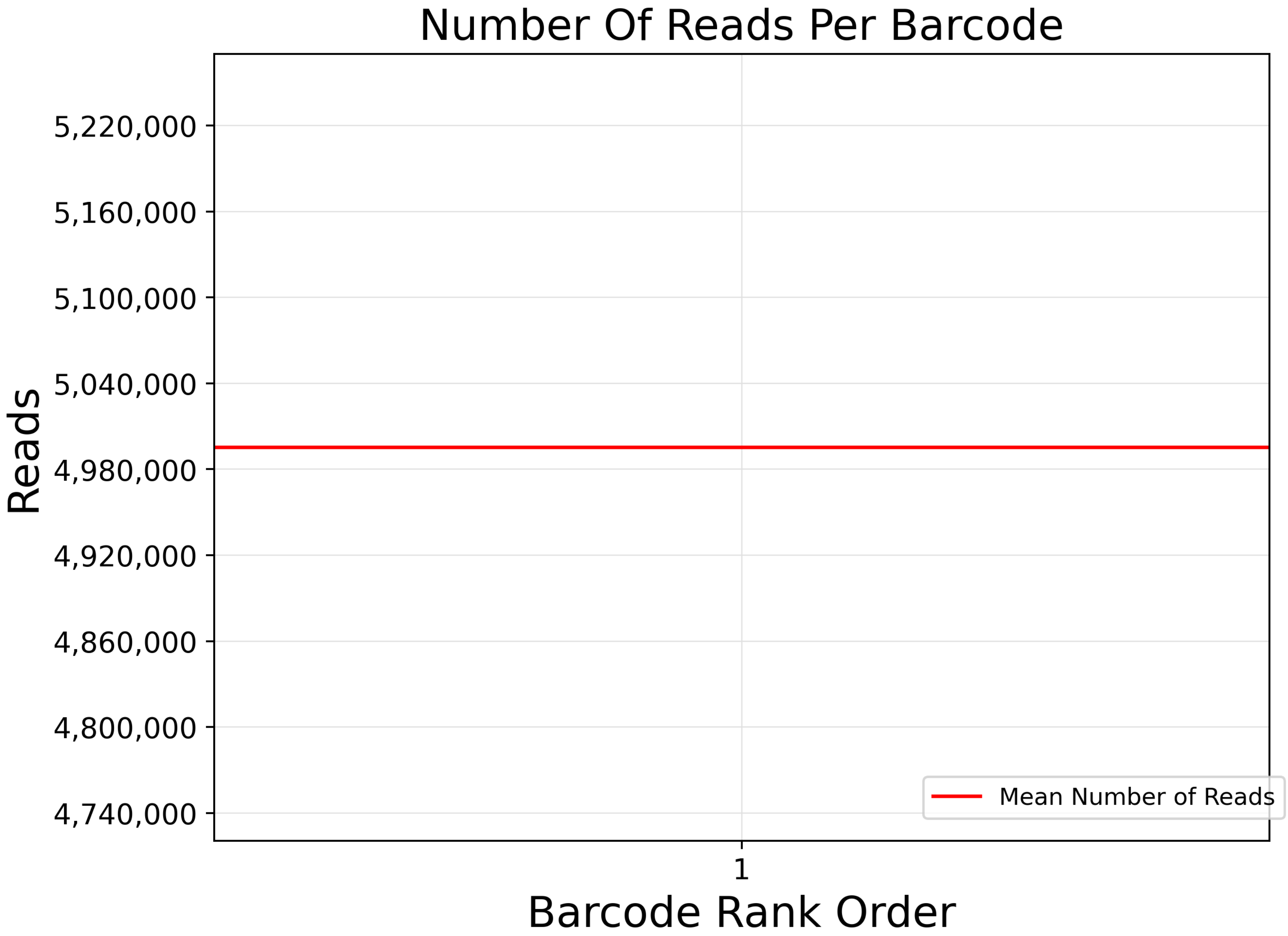

Barcode Frequency Distribution

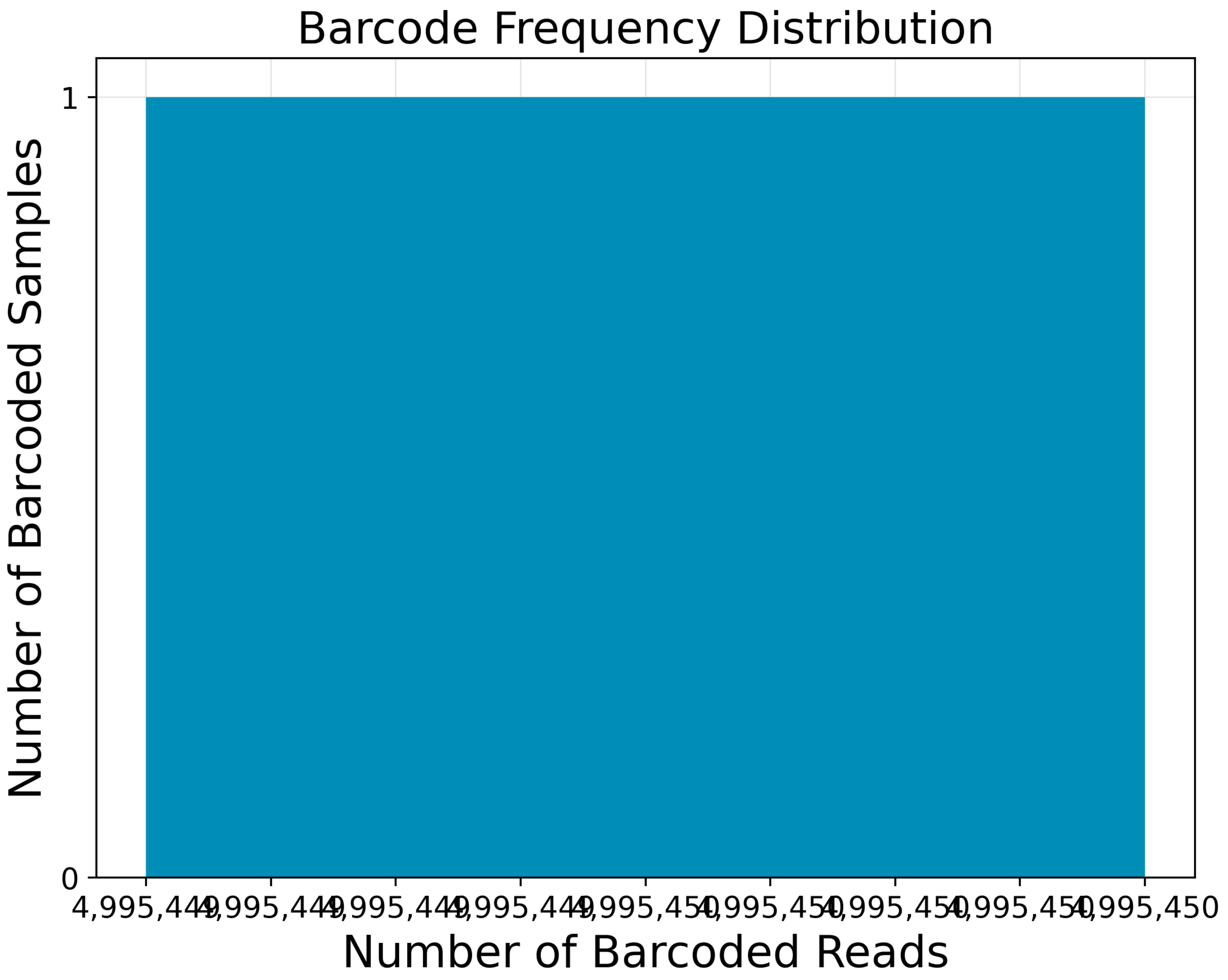

Barcode Frequency Distribution
Number of Barcoded Samples
Number of Barcoded Reads
1
0
4,995,449
4,995,449
4,995,449
4,995,449
4,995,450
4,995,450
4,995,450
4,995,450
4,995,450

Mean Read Length Distribution

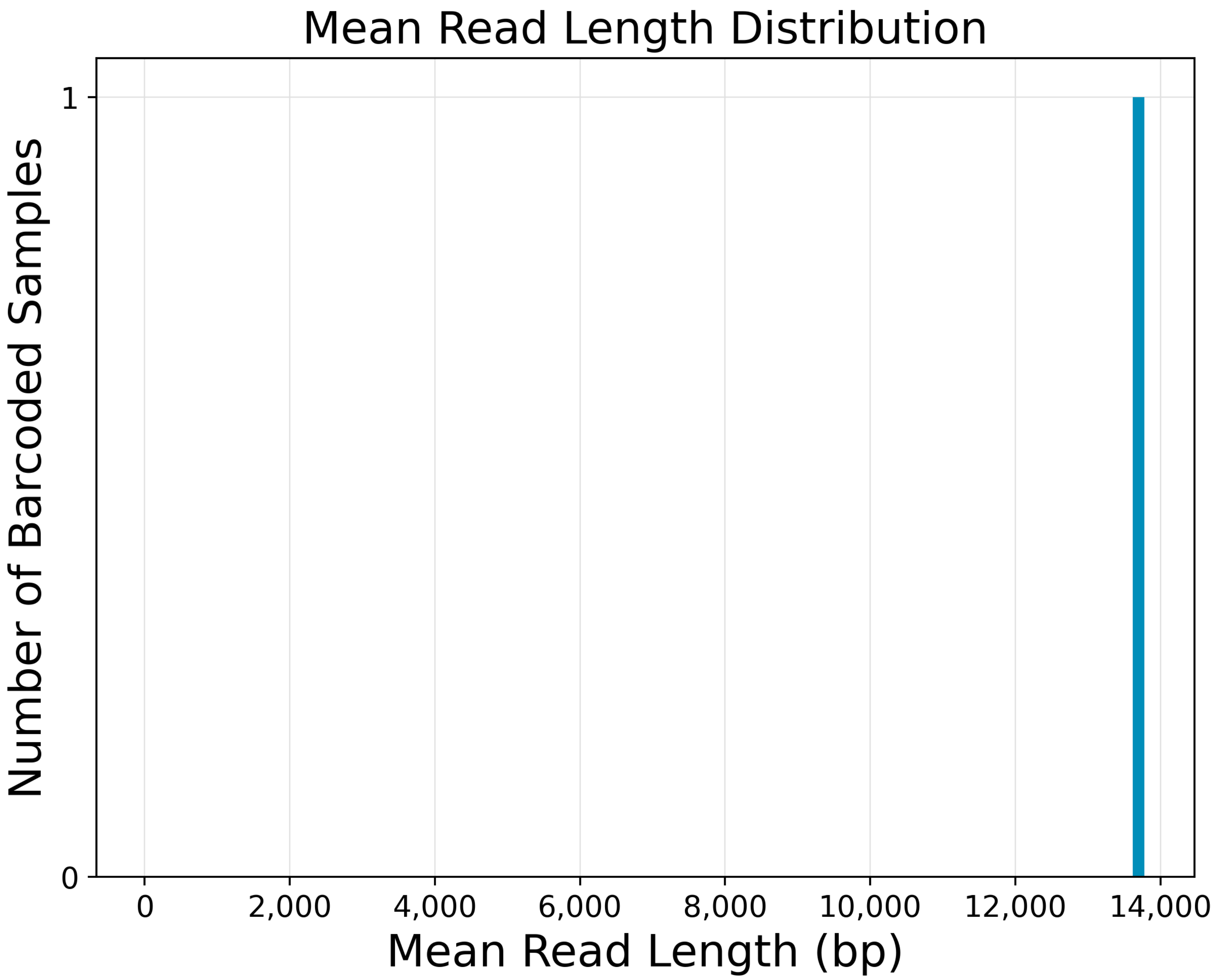

Mean Read Length Distribution
Number of Barcoded Samples
1
0
0
2,000
4,000
6,000
8,000
10,000
12,000
14,000
Mean Read Length (bp)

Barcode Quality Score Distribution

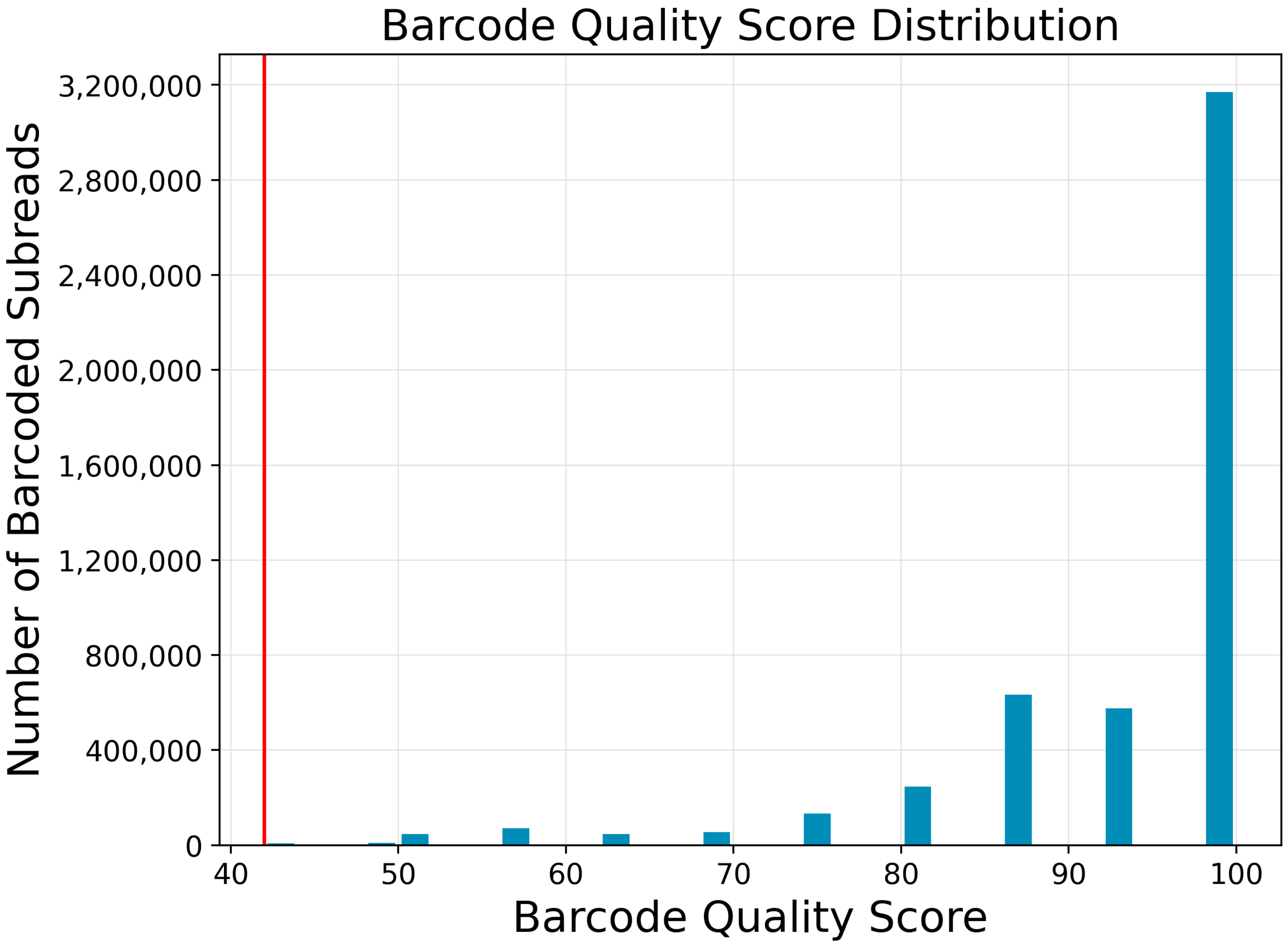

No Sample Setup found

Instrument run(s)

Run da66caee-c666-4192-91bb-d1f902e70baf

Summary

**Name**	Run 07.14.2023 18:18
**Status**	COMPLETE
**Created**	2023-07-15 00:27:39.656
**Started**	2023-07-15 00:28:50.790
**Completed**	2023-07-16 06:52:13.503
**Context**	r84100_20230715_002830
**Instrument Name**	84100
**Instrument Serial Number**	84100
**ICS Version**	12.0.0.179648
**Primary Analysis Version**	12.0.0.1
**Chemistry Version**	12.0.0.172289

Parent jobs (1)

Job 5644

Summary

**Job Type**	import-dataset
**Pipeline**	cromwell.workflows.sl_dataset_reports
**Name**	import-dataset
**Comments**	Description for job Import PacBio DataSet
**Created At**	2023-07-16 14:30:20.353
**SMRT Link Version**	12.0.0.177059

No child jobs found
