# Supplementary material for: A near chromosome-level genome assembly of a ghost moth (Lepidoptera, Hepialidae)
Source: Sci Data. 2024 Oct 16;11:1139. doi: 10.1038/s41597-024-03783-2 (PMC11484951; doi:10.1038/s41597-024-03783-2)
Supplement: Supplementary file 5 — Supporting Information [file 41597_2024_3783_MOESM5_ESM.docx]

**Supporting Information for**

**A near chromosome-level genome assembly of a ghost moth (Lepidoptera, Hepialidae)**

Yi-Ming Weng^1,2^, Isabel Lopez-Cacacho^1^, Bert Foquet^1^, Jose I. Martinez^1^, David Plotkin^1^, Andrei Sourakov^1^, Paul B. Frandsen^3^, Akito Y. Kawahara^1^

1. McGuire Center for Lepidoptera & Biodiversity, Florida Museum of Natural History, University of Florida, Gainesville, FL 32611, USA
2. Okinawa Institute of Science and Technology Graduate University, Okinawa, Japan
3. Department of Plant and Wildlife Sciences, Brigham Young University, Provo, Utah, USA

Corresponding author: Akito Y. Kawahara (kawahara@flmnh.ufl.edu)

Corresponding author: Akito Y. Kawahara ([kawahara@flmnh.ufl.edu](mailto:kawahara@flmnh.ufl.edu))

**Supplementary Table S1.** Summary of repeat elements in the *Druceiella hillmani* genome from RepeatMasker

| **Repeat elements** | **Family** | **Number of elements** | **Length occupied**  **(bp)** | **Percentage of sequence** |
| --- | --- | --- | --- | --- |
| SINEs |  | 0 | 0 | 0.00% |
|  | ALUs | 0 | 0 | 0.00% |
|  | MIRs | 0 | 0 | 0.00% |
| LINEs |  | 389,454 | 118,071,727 | 4.57% |
|  | LINE1 | 0 | 0 | 0.00% |
|  | LINE2 | 79,256 | 23,837,622 | 0.92% |
|  | L3/CR1 | 76,747 | 24,496,779 | 0.95% |
| LTR elements |  | 160,513 | 45,974,112 | 1.78% |
|  | ERVL | 0 | 0 | 0.00% |
|  | ERVL-MaLRs | 0 | 0 | 0.00% |
|  | ERV_classI | 0 | 0 | 0.00% |
|  | ERV_classII | 0 | 0 | 0.00% |
| DNA elements |  | 421,680 | 139,006,240 | 5.38% |
|  | hAT-Charlie | 5,337 | 2,685,584 | 0.10% |
|  | TcMar-Tigger | 3,713 | 1,437,454 | 0.06% |
| Unclassified |  | 2,277,069 | 457,102,022 | 17.68% |
| Total interspersed repeats |  |  | 760,154,101 | 29.39% |
| Small RNA |  | 6,858 | 1,454,523 | 0.06% |
| Satellites |  | 0 | 0 | 0.00% |
| Simple repeats |  | 772,641 | 47,649,454 | 1.84% |
| Low complexity |  | 115,212 | 5,573,392 | 0.22% |

**Supplementary Table S2.** Summary of the gene model from the Augustus prediction in BRAKER3 pipeline

| **Description** | **Statistics** |
| --- | --- |
| Number of genes | 61,160 |
| Number of monoexonic genes | 13,595 |
| Number of multiexonic genes | 47,527 |
| Number of positive strand genes | 30,085 |
| Number of positive strand monoexonic genes | 6,730 |
| Number of positive strand multiexonic genes | 23,317 |
| Number of negative strand genes | 31,075 |
| Number of negative strand monoexonic genes | 6,865 |
| Number of negative strand multiexonic genes | 24,210 |
| Average overall gene size | 7,667.15 |
| Median overall gene size | 3,187 |
| Average overall CDS size | 753.413 |
| Median overall CDS size | 435 |
| Average overall exon size | 218.838 |
| Median overall exon size | 165 |
| Average size of monoexonic genes | 497.332 |
| Median size of monoexonic genes | 318 |
| Largest monoexonic gene | 12,906 |
| Smallest monoexonic gene | 110 |
| Average size of multiexonic genes | 9,723.85 |
| Median size of multiexonic genes | 5407 |
| Largest multiexonic gene | 197,036 |
| Smallest multiexonic gene | 240 |
| Average size of multiexonic CDS | 827.267 |
| Median size of multiexonic CDS | 480 |
| Largest multiexonic CDS | 81,981 |
| Smallest multiexonic CDS | 21 |
| Average size of multiexonic exons | 199.616 |
| Median size of multiexonic exons | 159 |
| Average size of multiexonic introns | 2,829.23 |
| Median size of multiexonic introns | 1641 |
| Average number of exons per multiexonic gene | 4.144 |
| Median number of exons per multiexonic gene | 3 |
| Largest multiexonic exon | 14,886 |
| Smallest multiexonic exon | 3 |
| Most exons in one gene | 294 |
| Average number of introns per multiexonic gene | 3.144 |
| Median number of introns per multiexonic gene | 2 |
| Largest intron | 53,398 |
| Smallest intron | 16 |
| Number of complete models | 59,371 |
| Number of 5' only incomplete models | 997 |
| Number of 3' only incomplete models | 676 |
| Number of 5' and 3' incomplete models | 78 |


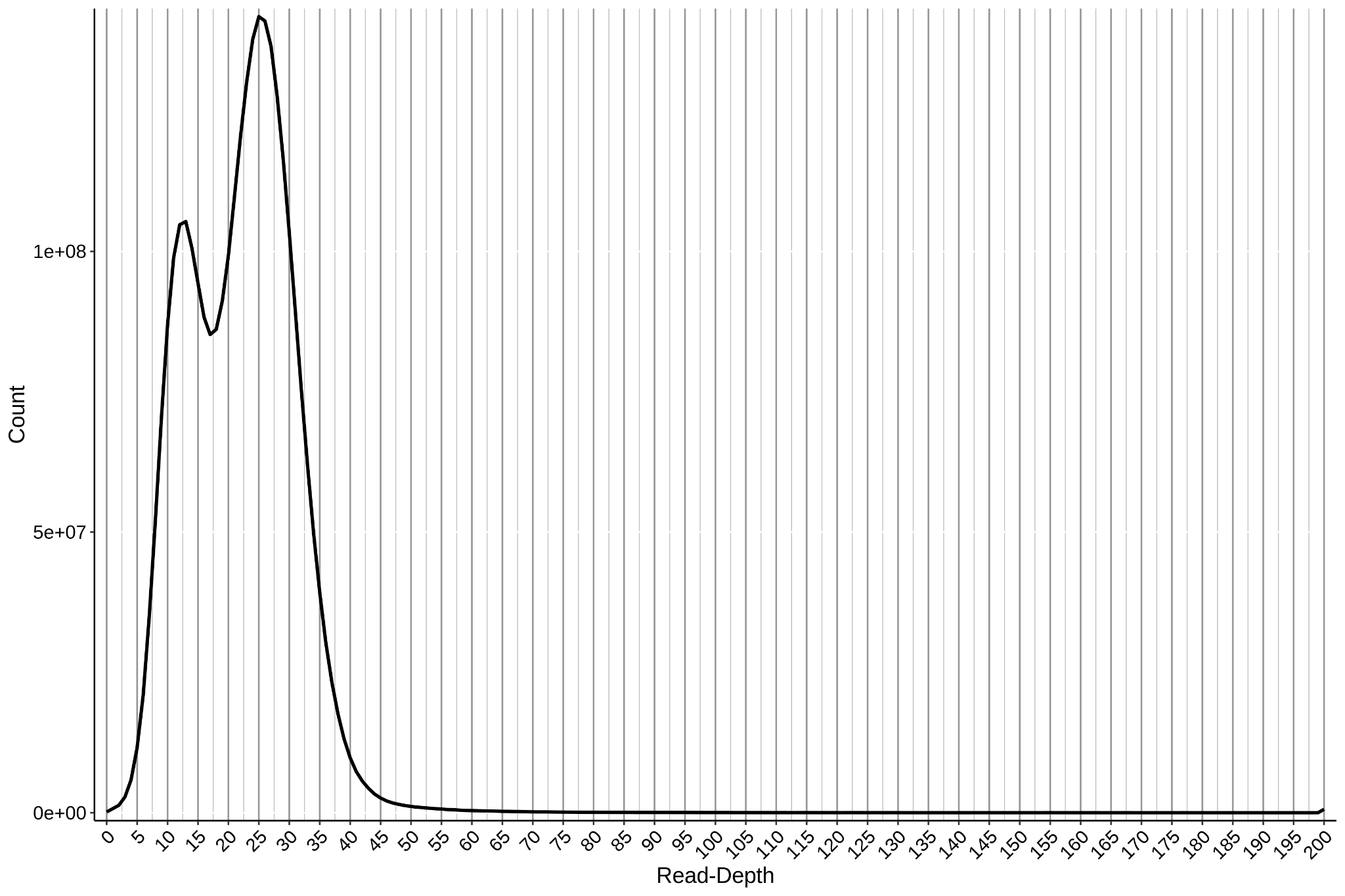


**Supplementary Figure S1.** Mapped coverage histogram generated from the purge_haplotigs pipeline. The read depth was calculated based on the mapping results of minimap2 where the raw sequence reads were mapped to the raw Hifiasm assembly.


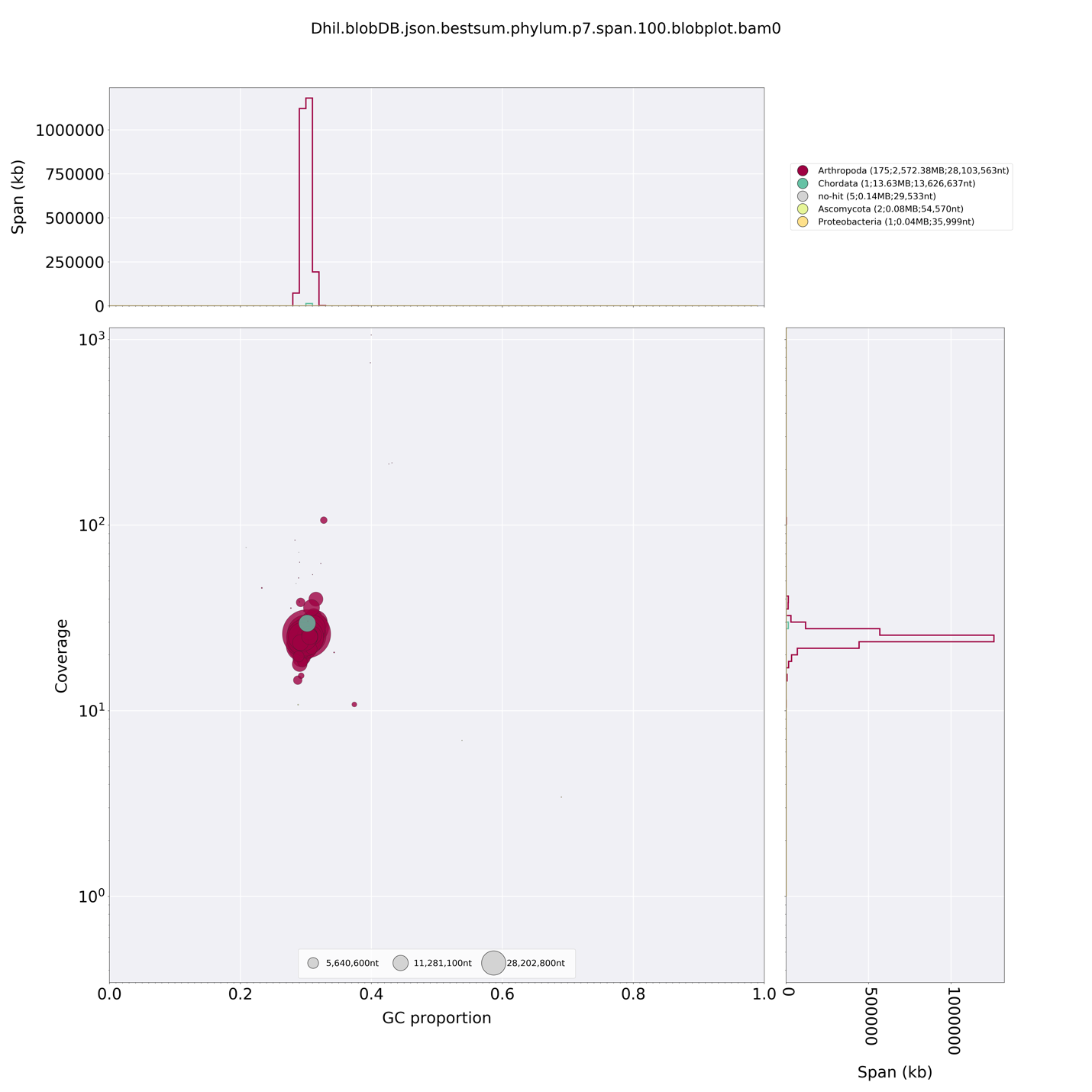


**Supplementary Figure S2.** BlobPlot of the *Druceiella hillmani* purged genome assembly. Red dots show contigs with best blast hits to Arthropoda; light green to Ascomycota; dark green dot Chordata; yellow dot to Proteobacteria; and gray dots had no hits. Contigs with best hit to Ascomycota and Proteobacteria were removed and the contig with best hit to Chordata was kept.


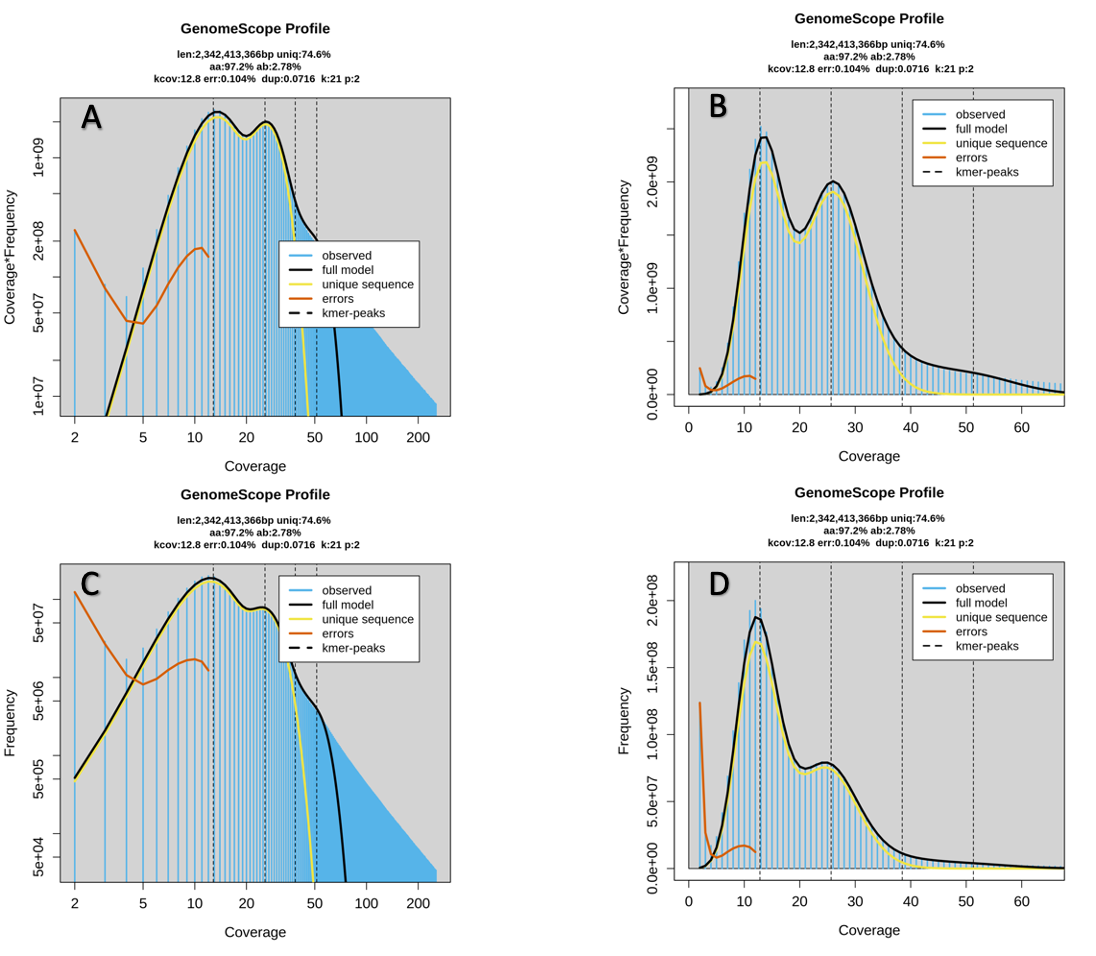


**Supplementary Figure S3.** K-mer distribution calculated using KMC with k-mer size of 21 bps.
The fitting model (black line) is created using GenomeScope2. The estimated genome size is around 2,342 Mb with k-mer coverage being 12.8X (homozygous peak coverage).
